# Combined MRI-Based Morphometric Analysis in Patients with Supraspinatus Tendon Tears: A Comparative Study

**DOI:** 10.3390/diagnostics16131990

**Published:** 2026-06-26

**Authors:** Gizem Kaya, Keziban Karacan, Alper Karacan, Mehtap Erdogan

**Affiliations:** 1 Department of Anatomy, Faculty of Medicine, Kocaeli University, 41380 Kocaeli, Türkiye; 54gizemkaya@gmail.com; 2 Department of Anatomy, Faculty of Medicine, Sakarya University, 54290 Sakarya, Türkiye; kkaracan@sakarya.edu.tr; 3 Department of Radiology, Faculty of Medicine, Sakarya University, 54290 Sakarya, Türkiye; alperkaracan@sakarya.edu.tr

**Keywords:** rotator cuff tear, magnetic resonance imaging, critical shoulder angle, supraspinatus tendon, acromiohumeral distance, shoulder morphometry

## Abstract

**Background/Objectives**: Rotator cuff tears (RCT) are one of the most common causes of shoulder pain and loss of function. The aim of this study was to evaluate shoulder morphometric and soft tissue parameters using magnetic resonance imaging (MRI) and to compare them between patients diagnosed with RCT and symptomatic controls without MRI-evident RCT. **Methods**: In this retrospective study, shoulder MRI images of 64 patients diagnosed with RCT and 63 control subjects without RCT aged 20–80 years were analyzed. The critical shoulder angle (CSA), lateral acromial angle (LAA), acromial index (AI), coracohumeral distance (CHD), acromiohumeral distance (AHD), coracoacromial ligament (CAL) thickness, and supraspinatus tendon (SST) thickness were measured. Binary logistic regression analysis was applied to identify independent factors associated with RCT. **Results**: CSA was significantly higher in the RCT group compared to the symptomatic control group without RCT (36.18 ± 4.0 vs. 32.61 ± 2.6, *p* < 0.001). In contrast, CHD (7.06 ± 1.13 vs. 8.05 ± 1.30 mm, *p* < 0.001), AHD (6.15 ± 1.27 vs. 7.23 ± 0.99 mm, *p* < 0.001), and SST thickness (5.17 ± 0.69 vs. 6.89 ± 0.64 mm, *p* < 0.001) were significantly lower in the RCT group. No significant differences were found between the groups in terms of LAA, AI, and CAL thickness (*p* > 0.05). In the logistic regression analysis, increasing age (OR = 1.159; *p* = 0.044) and CSA (OR = 1.808; *p* = 0.005) were identified as independent risk factors, while an increase in SST thickness was identified as inversely associated with RCT (OR = 0.35; *p* = 0.003). ROC analysis demonstrated that SST thickness had the highest discriminatory performance (AUC = 0.963), while the combined multivariable model achieved an AUC of 0.979. **Conclusions**: CSA, CHD, AHD, and SST thickness are important morphometric and soft tissue parameters associated with RCT. In particular, the assessment of CSA and SST thickness may provide a more robust and clinically meaningful approach to RCT-associated changes. The combined evaluation of CSA and SST thickness may contribute to the understanding of morphometric associations related to RCT.

## 1. Introduction

The shoulder joint possesses the greatest range of motion among all joints in the human body and relies on a complex interaction of osseous structures, muscles, tendons, and ligaments to maintain stability and function [1]. The rotator cuff, consisting of the supraspinatus, infraspinatus, teres minor, and subscapularis tendons, plays a critical role in dynamic stabilization of the glenohumeral joint by maintaining humeral head centering during shoulder motion [2]. Disruption of rotator cuff integrity may impair shoulder biomechanics, resulting in pain, weakness, functional limitation, and reduced quality of life [3].

The pathogenesis of rotator cuff tears (RCTs) is multifactorial and involves both intrinsic and extrinsic mechanisms. Intrinsic factors include age-related tendon degeneration, hypovascularity, collagen disorganization, oxidative stress, and reduced healing capacity. Extrinsic factors are mainly related to altered shoulder biomechanics, subacromial impingement, and anatomical variations that may increase mechanical loading on the rotator cuff tendons. Progressive tendon degeneration combined with repetitive mechanical stress is thought to contribute to tendon failure and tear formation [4,5].

Magnetic resonance imaging (MRI) plays a pivotal role in the evaluation of rotator cuff disorders since it allows comprehensive assessment of both osseous and soft tissue structures within a single examination. Compared with conventional radiography and ultrasonography, MRI provides superior visualization of tendon integrity, muscle atrophy, fatty degeneration, and associated morphometric characteristics without ionizing radiation. Therefore, MRI is considered the reference imaging modality for the comprehensive evaluation of rotator cuff pathology and related anatomical variations [6,7].

RCTs are among the most common causes of shoulder pain and dysfunction and represent a substantial healthcare burden worldwide. The prevalence of RCT increases markedly with age, with degenerative tears being particularly common in older adults [4]. In addition to causing pain and functional impairment, RCTs may negatively affect quality of life and daily activities, frequently leading to medical consultations and surgical interventions [3,8]. In addition to intrinsic degenerative processes, alterations in shoulder biomechanics are thought to play a significant role in the development and progression of RCTs. Anatomical variations in the acromion, scapula, and surrounding osseous structures may influence the distribution of mechanical loads across the rotator cuff tendons and contribute to subacromial impingement [4,9]. Progressive tendon degeneration and loss of rotator cuff integrity may result in superior migration of the humeral head, leading to narrowing of the subacromial space and increased mechanical stress on the supraspinatus tendon [4]. Consequently, several shoulder morphometric parameters have been investigated as potential anatomical factors associated with RCTs, including the critical shoulder angle (CSA), acromial index (AI), lateral acromial angle (LAA), acromiohumeral distance (AHD), and coracohumeral distance (CHD) [5,10,11].

Additionally, the relationship between soft tissue parameters such as the supraspinatus tendon (SST) and the coracoacromial ligament (CAL) and RCTs has been investigated; however, conflicting results have been reported in the literature regarding this topic [12,13]. In particular, it is thought that changes in tendon thickness may be related to both the degenerative process and the mechanism of the tear [7,14,15].

Although numerous studies have investigated individual morphometric parameters such as the critical shoulder angle (CSA), acromial index (AI), lateral acromial angle (LAA), acromiohumeral distance (AHD), and coracohumeral distance (CHD), these parameters have frequently been evaluated separately. Despite the growing number of studies investigating individual morphometric parameters, MRI-based studies integrating both osseous morphometry and tendon-related soft tissue characteristics within the same analytical model remain limited. Therefore, a combined assessment of morphometric and soft tissue parameters may provide a more comprehensive understanding of the multifactorial mechanisms associated with rotator cuff tears and represents the main rationale and novelty of the present study [7,10].

The aim of this study was to compare morphometric and soft tissue parameters—such as CSA, LAA, AI, CHD, AHD, CAL thickness, and SST thickness—measured from shoulder MRI images in patients with RCT and control groups, and to investigate the associations between these morphometric and soft tissue parameters and the presence of rotator cuff tears. Additionally, logistic regression analysis was performed to determine whether these variables are independent risk factors.

## 2. Materials and Methods

### 2.1. Study Design and Patient Population

This retrospective study was conducted by evaluating shoulder magnetic resonance images of patients who presented to the Orthopedics Clinic at Sakarya University Training and Research Hospital. The study included 64 patients (case group) aged 20–80 years who had been diagnosed with an RCT and 63 individuals (control group) who presented with shoulder pain but were found not to have an RCT.

This study was designed as a retrospective case–control study. However, the control group consisted of patients who had shoulder pain but no evidence of RCT on MRI. Therefore, these individuals should be considered a control group rather than truly healthy controls. This distinction is important since shoulder pain in the absence of RCT may still be associated with other shoulder pathologies, including rotator cuff tendinopathy, subacromial impingement, bursitis, labral lesions, adhesive capsulitis, or mild degenerative changes. These conditions may potentially influence morphometric measurements and SST thickness.

Patients with a history of fracture or infection, those who had previously undergone surgery on the same shoulder, and individuals with neurological deficits (e.g., hemiplegia) were excluded from the study. The RCT cases included in the study predominantly represented chronic degenerative supraspinatus tears rather than acute traumatic injuries.

Ethical approval for the study was obtained from the Ethics Committee of the Faculty of Medicine at Sakarya University via Decision No. 95 dated 28 March 2024.

### 2.2. Imaging Protocol

All MRI examinations were performed using a 1.5 Tesla scanner (Signa Voyager, GE Healthcare, Milwaukee, WI, USA). Patients were positioned in the supine position with the upper extremities in a neutral position.

Imaging parameters were standardized to obtain the following sequences: axial proton density fat-suppressed, coronal oblique T2-weighted fat-suppressed, coronal oblique T1-weighted, and sagittal oblique T2-weighted slices. The imaging matrix was set to 256 × 256, the field of view (FOV) to 16 cm, and the slice thickness to 3 mm.

### 2.3. Image Analysis and Measurements

All measurements were performed on standardized shoulder MRI images. Measurement parameters were evaluated in two main groups: (i) angular/morphometric measurements and (ii) distance/soft tissue measurements. CSA, LAA, AI, and AHD measurements were obtained from coronal oblique T1-weighted images. CHD was measured on axial proton density fat-suppressed images. CAL thickness was assessed on sagittal oblique T2-weighted images, whereas SST thickness was measured on coronal oblique T2-weighted fat-suppressed images. Measurements were standardized under the guidance of a radiologist experienced in shoulder MRI evaluation, and all morphometric measurements were performed by a single researcher. To assess intraobserver reproducibility, repeated measurements were performed on 20 randomly selected MRI examinations at different time points. Intraclass correlation coefficient (ICC) analysis was used to evaluate measurement reliability. During the measurement process, images were evaluated in random order, and care was taken to minimize systematic measurement errors.

#### 2.3.1. Angular and Morphometric Measurements

**Figure 1:**
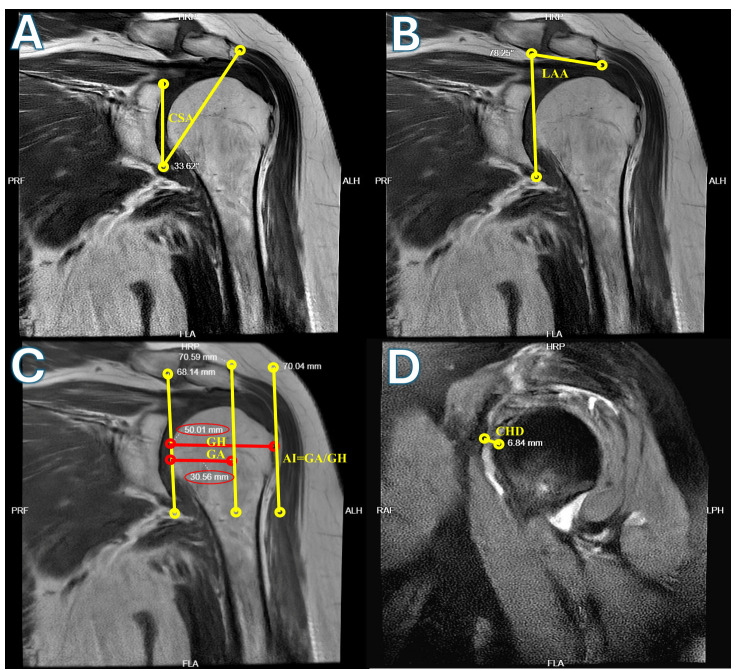
Angular and morphometric measurements on shoulder MRI. (**A**) Critical shoulder angle (CSA), measured as the angle formed between the glenoid line and the line connecting the inferior glenoid margin to the lateral edge of the acromion. (**B**) Lateral acromial angle (LAA), measured as the angle between the glenoid plane and the inferior surface of the acromion. (**C**) Acromial index (AI), calculated as the ratio of the lateral extension of the acromion to the lateral aspect of the humeral head, and acromiohumeral distance (AHD), defined as the shortest distance between the inferior surface of the acromion and the humeral head. Both measurements were obtained on a coronal oblique T1-weighted MRI image. (**D**) Coracohumeral distance (CHD), measured as the shortest distance between the coracoid process and the lesser tuberosity on an axial MRI image. HLP, ALH, PRF, and FLA represent orientation markers automatically generated by the MRI system.

Measurements of the CSA, LAA, AI, and CHD were determined using standard reference points (Figure 1).

CSA was defined as the angle formed between the inferior and superior margins of the glenoid and the lateral margin of the acromion. LAA was measured as the angle between a line parallel to the glenoid plane and a line parallel to the inferior surface of the acromion. AI was calculated as the ratio of the lateral projection of the acromion to the head of the humerus. CHD was assessed as the shortest distance between the coracoid process and the tuberculum minus.

#### 2.3.2. Distance and Soft Tissue Measurements

**Figure 2:**
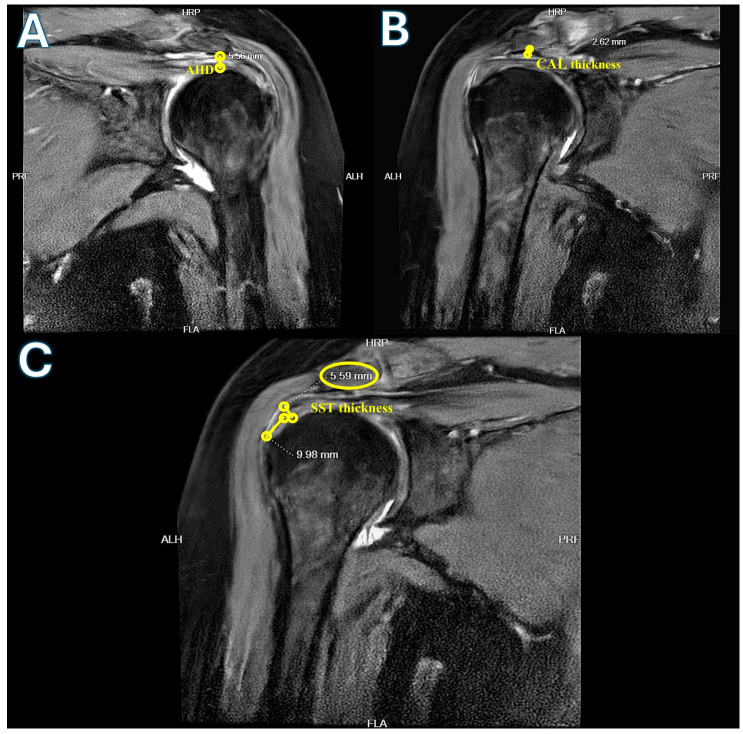
Distance and soft tissue measurements on shoulder MRI. (**A**) Acromiohumeral distance (AHD), defined as the shortest distance between the inferior surface of the acromion and the humeral head, measured on a coronal oblique T1-weighted MRI image. (**B**) Coracoacromial ligament (CAL) thickness measured at the thickest portion of the ligament on a sagittal oblique T2-weighted MRI image. (**C**) Supraspinatus tendon (SST) thickness measured approximately 10 mm lateral to the biceps tendon on a coronal oblique T2-weighted fat-suppressed MRI image. HLP, ALH, PRF, and FLA represent orientation markers automatically generated by the MRI system.

AHD, CAL thickness, and SST thickness were measured (Figure 2).

AHD was defined as the shortest distance between the inferior surface of the acromion and the head of the humerus. CAL thickness was measured at the thickest point of the ligament, whereas SST thickness was assessed at a distance of approximately 10 mm from the biceps tendon.

### 2.4. Evaluation of RCT

**Figure 3:**
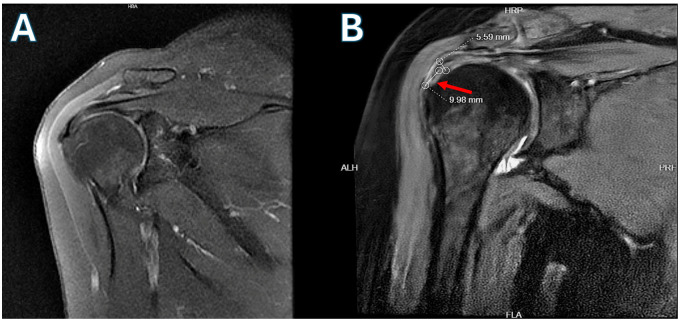
Representative MRI findings of the supraspinatus tendon (SST). (**A**) Normal SST demonstrating preserved tendon continuity and normal signal intensity. (**B**) Rotator cuff tear (RCT) showing disruption of tendon continuity with increased intratendinous signal intensity (arrows).

The diagnosis of an RCT was based on MRI findings. RCT diagnosis was primarily based on structural tendon abnormalities detected on MRI, including tendon discontinuity and/or visible tendon defects involving the SST. Increased signal intensity was evaluated together with these structural abnormalities and was not used as an isolated diagnostic criterion. The study predominantly included chronic degenerative SST tears. Partial- and full-thickness tears were evaluated together and were not analyzed separately due to the retrospective study design and limited subgroup sizes. The differences between normal tendon structure and the torn tendon were also visually compared (Figure 3). Detailed tear classification data, including tendon retraction grade, fatty degeneration, and muscle atrophy, were not consistently available in the retrospective MRI records.

### 2.5. Statistical Analysis

The data obtained were analyzed using SPSS version 23.0 (IBM Corp., Armonk, NY, USA). The normality of continuous variables was assessed using the Shapiro–Wilk test. Continuous variables with normal distribution were compared using the independent samples *t*-test, whereas non-normally distributed variables were compared using the Mann–Whitney U test. Categorical variables were analyzed using the chi-square test. Descriptive statistics were expressed as mean ± standard deviation. A *p*-value < 0.05 was considered statistically significant.

Receiver operating characteristic (ROC) curve analysis was performed to evaluate the discriminatory performance of individual morphometric parameters and the multivariable logistic regression model. Area under the curve (AUC), sensitivity, specificity, and optimal cut-off values based on the Youden index were calculated.

Binary logistic regression analysis was performed to identify independent risk factors associated with RCT. Age, CSA, LAA, AI, CHD, AHD, CAL thickness, and SST thickness were included in the model. Before model construction, multicollinearity among independent variables was assessed using tolerance and variance inflation factor (VIF) statistics. Tolerance values > 0.1 and VIF values < 5 were considered acceptable indicators of collinearity. Variables entered into the model were selected based on clinical relevance and the results of univariate analyses. To reduce the risk of overfitting, the number of variables included in the model was limited. Binary logistic regression analysis was performed using the enter method, and model fit was assessed using the Hosmer–Lemeshow goodness-of-fit test.

Due to the retrospective design of the study, an a priori sample size calculation was not performed. A post hoc power analysis was conducted using G*Power software (version 3.1.9.7; Heinrich-Heine-Universität Düsseldorf, Düsseldorf, Germany) based on the difference in CSA values between the groups.

Intraobserver ICC was assessed using repeated measurements of 20 randomly selected MRI examinations. Intraclass correlation coefficient (ICC) analysis was used to evaluate measurement reproducibility.

## 3. Results

### 3.1. Demographic Characteristics

A total of 127 individuals were included in the study, comprising 64 patients in the RCT group and 63 participants in the control group. No statistically significant differences were observed between the groups regarding age or sex distribution (Table 1). Demographic characteristics of the study population are summarized in Table 1.

### 3.2. Morphometric Parameters and Group Comparisons

Comparisons of morphometric and soft tissue parameters between the RCT and control groups are presented in Table 2. CSA was significantly higher in the RCT group than in the control group (*p* < 0.001). In contrast, CHD, AHD, and SST thickness were significantly lower in the RCT group (all *p* < 0.001). No statistically significant differences were observed between the groups regarding LAA, AI, or CAL thickness (all *p* > 0.05).

### 3.3. Sex-Based Subgroup Analysis

Sex-stratified analyses demonstrated that CSA was significantly higher in the RCT group than in the control group in both male and female participants. Similarly, CHD, AHD, and SST thickness were significantly lower in the RCT group for both sexes. No significant differences were observed for LAA, AI, or CAL thickness. Detailed subgroup analysis results are presented in Table 3.

### 3.4. Analyses by Age Group

Participants were stratified into two age groups (20–55 years and 56–80 years) for subgroup analyses. In the younger age group, CSA was significantly higher, whereas CHD, AHD, and SST thickness were significantly lower in the RCT group compared with controls. Similar findings were observed in the older age group, where CSA remained significantly higher, and CHD, AHD, and SST thickness remained significantly lower in the RCT group. No significant differences were observed for LAA, AI, or CAL thickness in either age subgroup. Detailed results are presented in Table 3.

### 3.5. Logistic Regression Analysis

The results of the multivariable logistic regression analysis are presented in Table 4. Increasing age (OR = 1.159, 95% CI: 1.004–1.338, *p* = 0.044) and higher CSA values (OR = 1.808, 95% CI: 1.200–2.722, *p* = 0.005) were identified as independent factors associated with the presence of RCT. Conversely, greater SST thickness was independently associated with a lower likelihood of RCT (OR = 0.35, 95% CI: 0.18–0.62, *p* = 0.003). The variables LAA, AI, CHD, AHD, and CAL thickness did not demonstrate independent associations with RCT in the multivariable model. Multicollinearity analysis demonstrated acceptable tolerance values ranging from 0.571 to 0.943 and VIF values ranging from 1.061 to 1.752, indicating no significant multicollinearity among the predictor variables. The Hosmer–Lemeshow goodness-of-fit test demonstrated excellent model calibration (χ^2^ = 0.342, df = 8, *p* = 1.000), indicating good agreement between observed and predicted outcomes. These findings suggest that the combined assessment of CSA and SST thickness may be clinically relevant in the evaluation of RCT.

### 3.6. ROC Analysis

ROC analysis demonstrated that SST thickness had the highest discriminatory performance for differentiating RCT from controls (AUC = 0.963, 95% CI: 0.937–0.989, *p* < 0.001). AHD and CHD showed moderate diagnostic performance, with AUC values of 0.764 (95% CI: 0.678–0.850, *p* < 0.001) and 0.734 (95% CI: 0.647–0.821, *p* < 0.001), respectively. LAA, AI, and CAL thickness demonstrated poor discriminatory performance (AUC values close to 0.50).

Based on Youden index analysis, the optimal cut-off value for SST thickness was approximately 6.0 mm. The optimal cut-off values for AHD and CHD were approximately 7.0 mm and 6.8 mm, respectively. For CSA, the optimal threshold was approximately 32°.

The multivariable logistic regression model demonstrated excellent diagnostic performance (AUC = 0.979, 95% CI: 0.957–1.000, *p* < 0.001). Using a probability threshold of 0.50, the model achieved a sensitivity of 96.8%, specificity of 95.3%, and overall classification accuracy of 96.1%.

Nevertheless, the very high AUC should be interpreted with caution due to the relatively limited sample size and the absence of external or internal validation procedures. Future studies including larger cohorts and independent validation datasets are warranted to confirm the robustness and generalizability of the model.

## 4. Discussion

In our study, we found that CSA, CHD, AHD, and SST thickness were significantly different in individuals with RCT compared to the control group. These findings are generally consistent with the current literature supporting the role of shoulder biomechanics, anatomical morphology, and tendon degeneration in rotator cuff pathology.

The finding that CSA values were significantly higher in individuals with RCT is consistent with numerous studies in the literature. Nakamura et al. and Gerlach et al. emphasized the strong association between increased CSA and RCT [10,16]. Similarly, Çağlar et al. reported significantly higher CSA values in patients with tears [17]. In a large-scale study by Lin et al., CSA values were reported to be higher than those observed in our cohort, which may be related to ethnic and morphological differences between study populations [18]. Furthermore, Gumina et al. demonstrated that CSA increases with age, particularly in older individuals [19]. Recent MRI-based investigations have further strengthened the association between increased CSA and rotator cuff pathology. Gulcu et al. demonstrated that larger CSA values were independently associated with degenerative rotator cuff tears on MRI, whereas Gerlach et al. identified increased CSA as a highly specific predictor of full-thickness tears [5,10]. These findings support the concept that greater lateral extension of the acromion may increase superiorly directed shear forces across the glenohumeral joint, thereby contributing to progressive tendon overload and degeneration.

The evaluated morphometric parameters may reflect different biomechanical and pathological aspects of rotator cuff disease. Parameters such as CSA and acromial morphology are thought to represent anatomical predisposition and altered mechanical loading patterns, whereas acromiohumeral and coracohumeral distances may be more closely associated with subacromial narrowing and impingement-related changes. In contrast, SST thickness likely reflects tendon-specific degenerative alterations rather than purely anatomical risk factors. Therefore, these measurements should not be interpreted as representing identical pathological mechanisms but rather as complementary parameters contributing to RCT development and progression.

When evaluated in terms of LAA, no significant difference was found between the case and control groups. Similarly, the literature contains conflicting results regarding the association between LAA and RCT. Studies conducted by Liu et al. and Maalouly et al. found no significant association between LAA and rotator cuff tears [20,21]. Klasan et al. reported that LAA may vary with age but has no strong association with gender [22]. These heterogeneous findings suggest that the utility of LAA as a standalone diagnostic marker may be limited.

In our study, no significant difference was found between groups in terms of AI. Although several studies have reported an association between AI and RCT [23], others have failed to demonstrate a significant relationship [21]. Variations in imaging modalities, measurement techniques, and population characteristics may contribute to these conflicting findings. Furthermore, age-related anatomical changes and humeral morphology may influence AI measurements, particularly in older populations [24].

Regarding CHD, a significant narrowing was observed in individuals with RCT. This finding is consistent with studies by Watson et al., who demonstrated an association between reduced CHD and rotator cuff pathology [25]. Although Aktaş et al. reported less consistent findings, significant differences were observed in both male and female subgroups in our study. Individual variations in coracoid morphology may partly explain these discrepancies [26].

The AHD findings were also largely consistent with previous reports. A significant reduction in AHD was observed in the RCT group. Similar findings have been reported by Kılıç et al. and Çağlar et al. [17,27]. Razmjou et al. suggested that AHD values below 6 mm may represent a clinically relevant threshold associated with rotator cuff pathology [28]. Although some patients in our cohort exhibited values above this threshold, our results suggest that AHD alone may not be sufficient as an isolated diagnostic criterion.

The concurrent alterations observed in CSA, CHD, and AHD suggest that these parameters may represent interconnected biomechanical phenomena rather than independent anatomical findings. Increased CSA has been associated with superior humeral migration and altered force distribution across the rotator cuff, whereas reductions in CHD and AHD may reflect progressive narrowing of the subacromial and subcoracoid spaces. Together, these changes may increase mechanical stress on the rotator cuff tendons and contribute to tear development and progression [4,11].

No significant difference in CAL thickness was found between the groups. Similarly, Watanabe et al. reported that CAL thickness was not significantly associated with RCT [13]. In contrast, Alraddadi et al. demonstrated a possible relationship between CAL morphology and rotator cuff pathology in cadaveric specimens [29]. Differences between cadaveric and imaging-based studies may explain these inconsistent findings.

In our study, SST thickness was significantly lower in the RCT group. This finding is consistent with the concept that progressive tendon degeneration is accompanied by structural alterations, including collagen disorganization, tendon thinning, reduced mechanical strength, and impaired healing capacity. Recent MRI-based studies have demonstrated that quantitative assessment of the supraspinatus tendon may provide valuable information regarding tendon integrity and disease severity. Ece et al. reported that advanced MRI techniques can detect compositional changes within the supraspinatus tendon that are associated with tendon pathology [7]. Similarly, Bedi et al. emphasized that tendon degeneration represents a dynamic biological process involving extracellular matrix disruption, cellular alterations, and progressive loss of tendon quality [4]. Therefore, reduced SST thickness may reflect not only the presence of a tear but also the cumulative effects of chronic tendon degeneration. From a clinical perspective, MRI-based evaluation of SST morphology may provide complementary information beyond simple tear detection and may contribute to a more comprehensive assessment of rotator cuff disease severity and progression [4,6,7].

Binary logistic regression analysis demonstrated that age, CSA, and SST thickness were independently associated with RCT. The combined assessment of CSA and SST thickness may provide stronger discriminatory ability than isolated parameter assessment in differentiating patients with RCT from the control group. This observation supports the concept that integrating osseous morphometric measurements with tendon-related soft tissue characteristics may improve understanding of the multifactorial mechanisms underlying rotator cuff disease. The reversal in the direction of association observed for AHD between univariate and multivariable analyses may reflect interactions among morphometric parameters after statistical adjustment, highlighting the importance of multivariable assessment rather than isolated parameter interpretation.

The contribution of this study to the literature lies in its MRI-based approach combining both osseous morphometric and soft tissue measurements in the evaluation of RCT. While CSA, CHD, AHD, and SST thickness demonstrated significant associations with RCT, the diagnostic contribution of LAA, AI, and CAL thickness appeared limited when evaluated individually. Differences among studies may reflect variability in imaging protocols, measurement methods, and population characteristics.

The additional ROC analyses demonstrated that the combined multivariable model provided substantially greater diagnostic performance than individual morphometric parameters. However, the exceptionally high AUC observed for the multivariable model should be interpreted cautiously since the model was developed and tested within the same cohort, and neither internal nor external validation procedures were performed. Consequently, the predictive performance observed in the present study may overestimate performance in independent populations. Future prospective studies with larger cohorts and external validation are warranted to confirm the robustness and generalizability of these findings.

### Limitations

This study has several limitations. First, its retrospective cross-sectional design limits the causal interpretation of the observed associations. Second, the control group consisted of symptomatic patients without MRI-evident RCT rather than asymptomatic healthy individuals, which may have influenced the specificity of the comparisons. Furthermore, tear-related characteristics such as tear size, partial- or full-thickness involvement, tendon retraction, fatty degeneration, muscle atrophy, and chronicity were not consistently available due to the retrospective design and therefore could not be analyzed separately. Since these pathological features may influence morphometric measurements and SST thickness, future studies incorporating more detailed tear characterization are warranted. Potentially relevant confounding variables, including body mass index, smoking status, occupational activity, diabetes mellitus, dominant arm, and trauma history, were also not systematically available. Finally, subgroup analyses were performed with relatively limited sample sizes, which may have reduced statistical power and increased the possibility of model overfitting.

## 5. Conclusions

This study suggests that evaluating shoulder morphometry in patients with RCT using multiple measurements rather than a single parameter may be more meaningful. According to the findings, the CSA, CHD, AHD, and SST thickness are significant parameters associated with RCT. In contrast, LAA, AI, and CAL thickness showed limited association in this study.

In particular, the combined evaluation of CSA and SST thickness may provide complementary information regarding RCT-associated morphometric alterations. However, larger-sample, prospective studies are needed to support the clinical application of these findings.

## Figures and Tables

**Table 1:** Demographic characteristics of the study population.

Variable	RCT Group (*n* = 64)	Control Group (*n* = 63)	*p*-Value
Age (years, mean ± SD)	48.9 ± 11.2	46.75 ± 10.6	0.270
Female, *n* (%)	32 (50%)	33 (52.4%)	0.931
Male, *n* (%)	32 (50%)	30 (47.6%)	-

Age comparisons were performed using the independent samples *t*-test. Sex distribution was compared using the chi-square test. RCT: Rotator cuff tear.

**Table 2:** Comparison of morphometric parameters between RCT and control groups.

Parameter	RCT Group (Mean ± SD)	Control Group (Mean ± SD)	*p*-Value
CSA (°)	36.18 ± 4.0	32.61 ± 2.6	<0.001 *
LAA (°)	79.32 ± 3.5	78.88 ± 4.2	0.541
AI	0.59 ± 0.08	0.58 ± 0.07	0.380
CHD (mm)	7.06 ± 1.13	8.05 ± 1.30	<0.001 *
AHD (mm)	6.15 ± 1.27	7.23 ± 0.99	<0.001 *
CAL thickness (mm)	2.58 ± 0.44	2.57 ± 0.39	0.859
SST thickness (mm)	5.17 ± 0.69	6.89 ± 0.64	<0.001 *

CSA: Critical shoulder angle; LAA: Lateral acromial angle; AI: Acromial index; CHD: Coracohumeral distance; AHD: Acromiohumeral distance; CAL: Coracoacromial ligament; SST: Supraspinatus tendon (The data are presented as mean ± standard deviation. Comparisons between groups were performed using either an independent samples *t*-test or the Mann–Whitney U test, depending on the data distribution. * *p* < 0.05 was considered statistically significant).

**Table 3:** Subgroup analyses according to sex and age groups.

(A) Sex-Based Subgroup Analysis						
Parameter	Male RCT Mean ± SD	Male Control Mean ± SD	*p*-Value	Female RCT Mean ± SD	Female Control Mean ± SD	*p*-Value
CSA	36.44 ± 3.19	33.61 ± 2.89	0.001	35.92 ± 4.87	31.66 ± 2.75	<0.001
LAA	79.59 ± 3.88	79.21 ± 4.38	0.715	79.05 ± 4.07	78.57 ± 3.99	0.628
AI	0.60 ± 0.08	0.58 ± 0.07	0.309	0.59 ± 0.09	0.58 ± 0.08	0.786
CHD	7.31 ± 1.22	8.23 ± 1.41	0.008	6.82 ± 1.00	7.88 ± 1.19	<0.001
AHD	6.30 ± 1.26	7.59 ± 1.00	<0.001	6.00 ± 1.29	6.90 ± 0.87	0.002
CAL thickness	2.52 ± 0.33	2.57 ± 0.35	0.603	2.65 ± 0.52	2.58 ± 0.43	0.558
SST thickness	5.22 ± 0.72	6.95 ± 0.62	<0.001	5.11 ± 0.67	6.83 ± 0.66	<0.001
**(B) Age-Based Subgroup Analysis**						
**Parameter**	**Younger RCT Mean ± SD**	**Younger Control Mean ± SD**	***p*** **-Value**	**Older RCT Mean ± SD**	**Older Control Mean ± SD**	***p*** **-Value**
CSA	36.84 ± 3.81	32.70 ± 3.10	0.001	35.93 ± 4.22	32.44 ± 2.76	<0.001
LAA	79.65 ± 3.20	78.86 ± 4.38	0.451	79.20 ± 4.22	78.91 ± 3.83	0.778
AI	0.63 ± 0.09	0.59 ± 0.08	0.127	0.58 ± 0.08	0.57 ± 0.06	0.579
CHD	6.95 ± 1.08	7.84 ± 1.23	0.010	7.11 ± 1.16	8.42 ± 1.36	<0.001
AHD	6.33 ± 1.38	7.29 ± 0.87	0.015	6.08 ± 1.24	7.13 ± 1.19	0.001
CAL thickness	2.50 ± 0.54	2.64 ± 0.37	0.337	2.62 ± 0.39	2.45 ± 0.41	0.114
SST thickness	5.16 ± 0.78	6.92 ± 0.62	<0.001	5.17 ± 0.66	6.84 ± 0.68	<0.001

Comparisons between groups were performed using the independent samples *t*-test or Mann–Whitney U test according to data distribution. CSA: Critical shoulder angle; LAA: Lateral acromial angle; AI: Acromial index; CHD: Coracohumeral distance; AHD: Acromiohumeral distance; CAL: Coracoacromial ligament; SST: Supraspinatus tendon.

**Table 4:** Multivariable logistic regression analysis of parameters associated with RCT.

Variable	OR (Exp(B))	95% CI	*p*-Value
Age	1.159	1.004–1.338	0.044 *
CSA	1.808	1.200–2.722	0.005 *
LAA	1.254	0.946–1.662	0.115
AI	1.12	0.68–1.85	0.658
CHD	0.564	0.229–1.389	0.213
AHD	1.761	0.609–5.092	0.296
CAL thickness	1.08	0.72–1.61	0.703
SST thickness	0.35	0.18–0.62	0.003 *

CSA: Critical shoulder angle; LAA: Lateral acromial angle; AI: Acromial index; CHD: Coracohumeral distance; AHD: Acromiohumeral distance; CAL: Coracoacromial ligament; SST: Supraspinatus tendon CI: Confidence interval. Multicollinearity diagnostics demonstrated VIF values ranging from 1.061 to 1.752 and tolerance values ranging from 0.571 to 0.943. Hosmer–Lemeshow goodness-of-fit test: χ^2^ = 0.342, df = 8, *p* = 1.000. * *p* < 0.05 was considered statistically significant.

## Data Availability

The original contributions presented in this study are included in the article. Further inquiries can be directed to the corresponding author.

## Abbreviations

The following abbreviations are used in this manuscript:

RCT	Rotator cuff tears
MRI	Magnetic resonance imaging
CSA	Critical shoulder angle
CHD	Coracohumeral distance
AI	Acromial index
CAL	Coracoacromial ligament
SST	Supraspinatus tendon
